# Patterns of Morbidity in Ambatoboeny District, Northern Madagascar: A 12-Month Study

**DOI:** 10.3390/jcm14176329

**Published:** 2025-09-08

**Authors:** Daniel Kasprowicz, Krzysztof Korzeniewski, Wanesa Wilczyńska

**Affiliations:** 1 Clinique Medicale Beyzym, Ambatoboeny District, Manerinerina 403, Madagascar; daniel.kasprowicz@icloud.com; 2 Department of Epidemiology and Tropical Medicine, Military Institute of Medicine–National Research Institute, 04-141 Warsaw, Poland; kkorzeniewski@wim.mil.pl

**Keywords:** morbidity, Madagascar, infectious diseases, parasitic diseases

## Abstract

**Background:** Ambatoboeny District in northern Madagascar faces significant health challenges due to widespread poverty, poor access to healthcare, and limited diagnostic capabilities. Despite high disease burden, data on morbidity patterns in the region are scarce. This study aims to identify the most prevalent diseases and most affected demographic groups, thus providing valuable insight into the region’s health profile. **Methods**: A retrospective analysis was conducted on medical records from 3678 patients who were admitted at Clinique Médicale BEYZYM, a secondary-level referral facility in Manerinerina, Boeny Region between January and December 2024. Diagnoses were retrieved from physician registration ledgers, hospitalization records, monthly laboratory reports, monthly general hospital activity reports and monthly reports *from Centre de Traitement et de Diagnostic de la Tuberculose*, which were cross-referenced and verified by trained clinical staff. Records were included if they contained identifiable demographic data and at least one clinical diagnosis. Diagnoses were coded using ICD-11 and were classified into 15 major categories. **Results**: The median patient age was 19.5 years (IQR: 7–42), with females accounting for 54% of the cohort. Most patients (87.2%) resided in Ambatoboeny. The most common reasons for admission were infectious and parasitic diseases (35.75%, 95% CI: 34.20–37.30), respiratory diseases (22.73%, 95% CI: 21.38–24.08), and diseases of the genitourinary system (13.95%, 95% CI: 12.83–15.07), collectively accounting for 72.43% of all recorded cases. Statistically significant differences in morbidity patterns were observed across age and sex groups. **Conclusions**: The findings underscore the multifaceted burden of disease in the Ambatoboeny District, where both infectious and chronic conditions coexist in a resource-limited setting. Delayed healthcare-seeking behavior, cultural beliefs, and diagnostic limitations further complicate care delivery. This study provides foundational data to inform targeted health policies, humanitarian medical missions, and diagnostic capacity-building tailored to local needs.

## 1. Introduction

Madagascar remains one of the poorest countries in the world, with statistics indicating that in 2023 approximately 80.7% of the population lived below the international poverty line of 2.15 USD per day. In August 2024, inflation on the island reached 7.8%, driven primarily by surging food and energy prices. This economic pressure has exacerbated the ongoing crisis, contributing to a worsening hunger situation across the country and further limiting access to basic services [1]. In 2021, the Malagasy government allocated only 3.5% of its GDP to health expenditures, despite a population of over 31 million. This low investment highlights the country’s heavy reliance on external humanitarian assistance to sustain its healthcare system [2].

Madagascar is currently facing a complex public health crisis resulting from the dual burden of infectious and non-communicable diseases (NCDs). This phenomenon, poses unique challenges to the national healthcare system, necessitating an integrated approach to prevention, diagnosis, and treatment [3]. Among infectious diseases, malaria remains one of the most significant threats. In 2022, more than 3.56 million cases of malaria were reported, and the disease resulted in 547 deaths. The increase in incidence was 25–55% higher compared to 2015. These data underscore that malaria continues to pose a major public health challenge, despite global progress in malaria control [4]. Another major concern is tuberculosis (TB), which continues to affect the population at high rates. In 2023, the TB burden was estimated at 233 cases per 100,000 population (73,000 new TB cases), with 12,000 deaths among HIV-negative individuals and 720 deaths among people living with HIV. TB incidence among people with HIV was 6.8 per 100,000, and approximately 1100 cases of MDR/RR-TB were reported. Despite progress in control efforts, treatment coverage reached only 64%, while 75% of TB-affected households experienced catastrophic costs due to the disease. Closely associated with poverty, overcrowding, and undernutrition, TB remains a persistent threat, and its high prevalence highlights the need for more effective strategies in diagnosis, treatment, and prevention [5]. The situation is further complicated by the burden of HIV/AIDS. In 2023, there were approximately 90,000 people were living with HIV in Madagascar, with an uncertainty range between 69,000 and 120,000. Adult HIV prevalence among those aged 15–49 years was 0.5% [0.4–0.6], yet new infections remain a concern, with approximately 16,000 (12,000–22,000) individuals newly infected and around 3100 (2100–4600) deaths reported in 2023. This low level of awareness and treatment access significantly hampers efforts to control the epidemic and contributes to ongoing transmission [6]. Madagascar is also considered endemic for neglected tropical diseases (NTDs) The most relevant challenges include taeniasis and cysticercosis, schistosomiasis, leprosy, and chikungunya, which together exert a disproportionate impact on the most vulnerable groups. Although these diseases rarely result in direct mortality, they impose a substantial burden on health and quality of life, particularly among children and populations living in extreme poverty. In 2022, approximately 1.42% of the population required interventions against NTDs, underscoring persistent gaps in prevention and control measures [7]. At the same time, non-communicable diseases such as cardiovascular diseases, cancers, diabetes, and chronic respiratory diseases are becoming increasingly prominent. Since 2015, the age-standardized mortality rate due to NCDs has exceeded 600 deaths per 100,000 population, indicating a rapid epidemiological transition that is not yet matched by a corresponding development in healthcare infrastructure and capacity. Trends in childhood disease prevention are equally alarming and warrant immediate attention [3]. Childhood immunization coverage in Madagascar remains critically low. Recent analyses show that only 48.9% of children are fully vaccinated, far below the WHO target of 90% for the Expanded Programme on Immunization (EPI) [8]. In 2021, only 39% of children received the first dose of measles vaccine, and just 55% completed the full DTP series (diphtheria, tetanus, pertussis). Moreover, an estimated 303,775 children remained entirely unvaccinated, increasing the risk of outbreaks of vaccine-preventable diseases. All these factors contribute to alarming child mortality indicators. In 2021, under-five mortality stood at 66 per 1000 live births, and neonatal mortality at 24.1 per 1000 live births—significantly exceeding the targets set under the Sustainable Development Goals (SDGs) and highlighting the urgent need for strengthened public health interventions [3]. The situation is particularly critical in the Boeny and Sofia regions, where high rates of communicable diseases, inadequate access to healthcare, and underdeveloped health infrastructure pose serious risks to public health. However, detailed epidemiological data from these areas remain limited, complicating efforts to design and implement effective health interventions.

The aim of this study is to identify the morbidity profile, demographic gap, and overall health burden among residents of the Ambatoboeny district, located in the Boeny region of Madagascar. Based on data from consultations and hospitalizations recorded at the BEYZYM Clinic in Manerinerina, the study highlights the most prevalent diseases and outlines the major health challenges faced by physicians working in humanitarian missions. This analysis aims to provide essential data for the development of effective health interventions and the formulation of policies tailored to local needs. Considering the region’s demographic and epidemiological specificities is crucial for improving healthcare system efficiency and enhancing access to medical services for the population.

## 2. Materials and Methods

### 2.1. Study Setting, Medical Facility and Diagnostic Procedures

**Figure 1:**
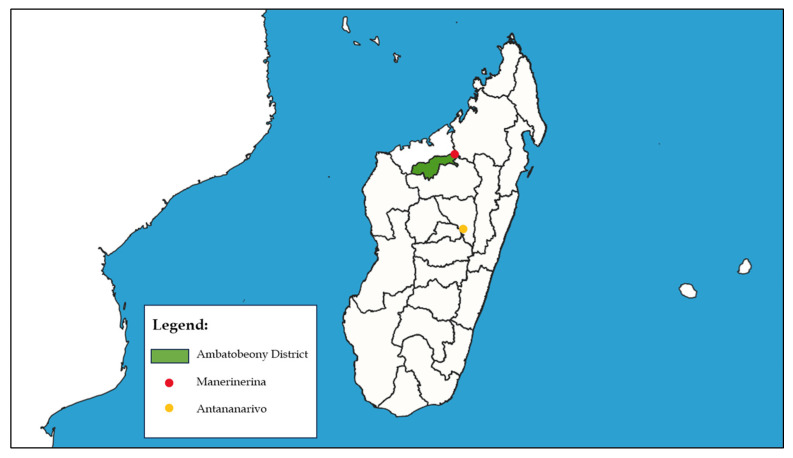
Location of the Ambatoboeny District in Madagascar.

This study was conducted at Clinique Médicale BEYZYM (accredited by the Ministry of Health under ARRETE No. 27 821/2024-MSANP), a secondary-level referral hospital in the Ambatoboeny District, located in Manerinerina (16°17′ S, 47°17′ E), Boeny Region, Madagascar. The geographical location of the Ambatoboeny district is illustrated in Figure 1. The clinic consists of several departments: pediatrics, multi-profile internal medicine, infectious diseases, and surgery. It also operates specialized units, including the Nutritional Rehabilitation and Education Centre (Centre de Récupération et d’Éducation Nutritionnelle–CREN), and the Tuberculosis Diagnosis and Treatment Centre (Centre de Diagnostic et de Traitement de la Tuberculose–CDT).

The hospital laboratory is certified by the Ministry of Health of Madagascar and supervised by a medical biologist who performs routine verification and validation of test results. The laboratory is equipped for hematological, biochemical, microbiological, parasitological, and serological testing.

Hematological analyses are performed using a Mindray M-30 hematology analyzer, with complementary manual blood smears stained using the May–Grünwald method. Biochemical assays are conducted with a Sinnowa BS-3000 analyzer.

Serological tests include immunochromatographic assays such as detection of hepatitis B surface antigen (HBsAg), antibodies against hepatitis C virus (anti-HCV), antigens of HIV-1 and HIV-2, troponin, prostate-specific antigen (PSA), antibodies against Helicobacter pylori, and antibodies for typhoid fever. Agglutination tests include detection of C-reactive protein (CRP), antistreptolysin O (ASO), rheumatoid factor (RF), and the Widal–Felix reaction for *Salmonella typhi*. Serological tests for syphilis, including *Treponema pallidum* hemagglutination assay (TPHA) and rapid plasma reagin (RPR), as well as the modified agglutination test (MAT) for detection of IgG antibodies against *Toxoplasma gondii*, are also used.

Additional diagnostic procedures include the Emmel test for detection of sickle cell anemia. Malaria diagnostics consist of rapid diagnostic tests for *P. falciparum* and pan-*Plasmodium* antigens (Pf/Pan Ag), as well as thin and thick blood smear microscopy. Coagulation analyses are conducted using a semi-automated coagulometer (Diagon Coag 2D).

For intestinal parasitic infections, stool samples are examined using direct smear preparations stained with Lugol’s iodine solution (KAU test). This method enables detection of helminth eggs (e.g., *Ascaris lumbricoides, Hymenolepis nana*) but has limited sensitivity for protozoal infections. Due to the lack of concentration techniques, immunoassays, or molecular diagnostics, protozoa could not always be identified at the species level. In such cases, laboratory records were limited to general observations (e.g., “protozoa” or “*Entamoeba* sp.”). For consistency, these cases were classified as gastroenteritis of infectious origin (ICD-11: 1A00–1A0Z) based on clinical findings and available laboratory data.

All infectious and parasitic diseases included in the analysis were classified only after confirmation by appropriate laboratory diagnostic methods. In addition, certain basic tests—such as complete blood count and urinalysis (for *Schistosoma haematobium*)—were routinely performed in all patients, regardless of their initial symptoms.

**Figure 2:**
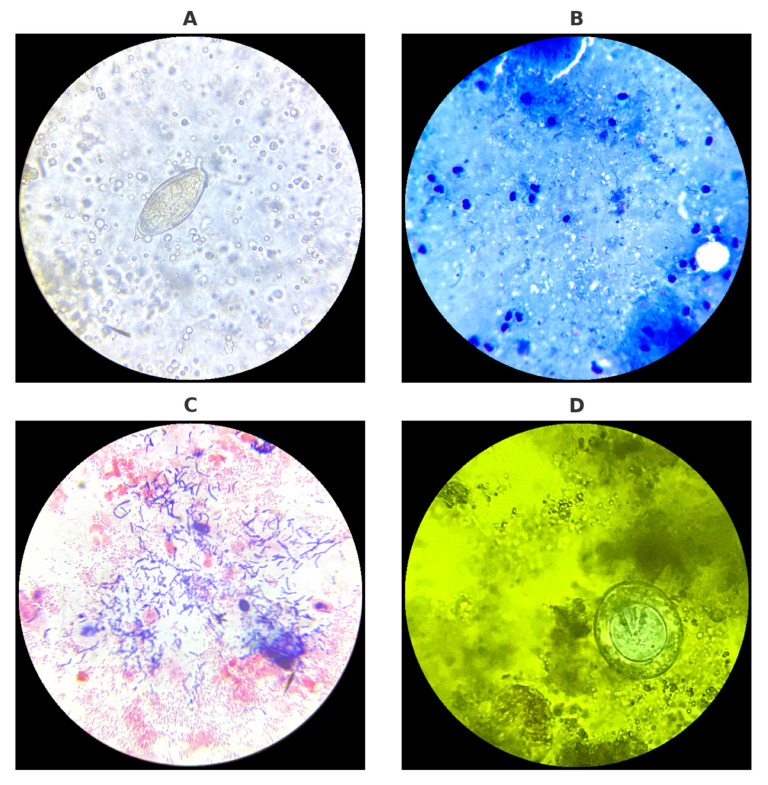
Microscopic diagnostics of urine, sputum, vaginal swab, and stool samples performed in the laboratory: (**A**) Egg of *Schistosoma haematobium* detected in urine sediment. (**B**) Acid-fast bacilli (*Mycobacterium tuberculosis*) visualized in Ziehl–Neelsen-stained sputum smear. (**C**) Gram-stained vaginal swab smear showing local bacterial microflora. (**D**) Egg of *Hymenolepis nana* observed in stool sample stained with Lugol’s iodine solution. Source: Kasprowicz D.

Representative microscopic images obtained during routine diagnostics are presented in Figure 2.

### 2.2. Demographic and Epidemiological Context

In 2020, Ambatoboeny District, located in the Boeny Region of northwestern Madagascar, had a projected population of 283,840, spread across an area of 8528 km^2^, resulting in a population density of approximately 33 persons per km^2^. According to the 2018 census, the gender distribution in the district was nearly equal, with 132,238 males and 131,932 females. The rural municipality of Manerinerina, where the study was conducted, had a population of 26,675 in 2018. The local economy is predominantly agrarian, with 50% of the population engaged in farming and 45% in livestock raising; rice is the principal crop [9]. Access to healthcare is limited, and Clinique Médicale BEYZYM serves as the primary referral centre for both Manerinerina and surrounding areas, including parts of the neighbouring Mampikony district.

### 2.3. Study Population and Data Sources

This retrospective observational study covered the period from 1 January 2024 to 31 December 2024. Medical data were retrieved from the following institutional sources: physician registration ledgers, hospitalization records, monthly laboratory reports, monthly general hospital activity reports and monthly reports from CDT. Patients were eligible for inclusion if their medical records contained identifiable demographic information (age and sex) and at least one documented diagnosis or symptomatic description. The following categories were excluded: records lacking core demographic or clinical data, follow-up (control) visits, patients who were dead on arrival, patients triaged and referred to a primary-level facility without a diagnosis being established. After applying the inclusion and exclusion criteria, a total of 3.678 patient records were retained for analysis. All data were anonymised prior to processing and analysis.

### 2.4. Diagnostic Classification

All diseases and conditions were classified using the International Classification of Diseases, 11th Revision (ICD-11) [10]. Diagnostic coding was performed in collaboration with two in-house physicians, and in cases of disagreement or uncertainty, a third opinion was obtained from an external medical professional. The classification of infectious diseases was additionally supported by the Équipe de Management de Région under the Direction Régionale de la Sécurité Publique (Mahajanga). For the purpose of statistical analysis, diseases were grouped into 15 major ICD-11 categories, including both system-based and general symptom-related classifications. This selection was guided by clinical relevance, the range of diagnostic capabilities available at the clinic, and the actual distribution of recorded morbidity patterns. Several ICD-11 categories were excluded from the analysis due to either the absence of corresponding cases in the study data or limitations in the hospital’s diagnostic and treatment capabilities. A summary of the excluded categories and the rationale for their exclusion is presented in Table 1.

### 2.5. Ethical Considerations

Ethical approval for this retrospective study was obtained from the District Public Health Office (Service du District de la Santé Publique—SDSP, Ambatoboeny) in September 2023 and from the Hospital Supervisory Committee of Clinique Médicale BEYZYM in July 2024. Patient confidentiality was strictly maintained, and all records were processed in an anonymized format to ensure compliance with ethical standards for retrospective medical research.

### 2.6. Data Analysis

Descriptive and inferential statistical analyses were performed using Apple Numbers (Version 6.1). The data were categorized by age groups (0–4, 5–14, 15–24, 25–59, 60+) and sex (male and female), and frequency distributions were generated for these demographic variables, as well as disease categories. To assess the relationship between sex and morbidity, a chi-squared test of independence was conducted. Cramér’s V was used to measure the strength of the association between sex and disease categories. Additionally, to evaluate the potential effect of age on morbidity, a Spearman’s rank correlation test was applied, treating age as an ordinal variable with the four predefined age intervals. To determine the five most prevalent disease categories in the overall population and within age groups, sex-specific conditions (e.g., pregnancy-related disorders and female pelvic inflammatory disease) were excluded from the general and age-stratified analyses, as their inclusion could bias the results due to their inherent restriction to one sex. These conditions were, however, retained in sex-specific analyses. Additionally, nonspecific symptom-based classifications (ICD-10 codes R00–R99: General symptoms and signs) were omitted from all prevalence analyses, as they do not represent definitive disease diagnoses.

For sex-stratified analyses, we excluded the ICD-11 chapter Pregnancy, childbirth and the puerperium to avoid biassing female morbidity with non-disease obstetric states. All other diagnoses—including female reproductive tract infections and sexually transmitted infections—were included under their respective ICD-11 chapters. No cases of pregnancy-associated malaria were recorded during the study period. Low birthweight was not treated as a morbidity diagnosis and was therefore excluded. Routine antenatal consultations were not counted as morbidity; pregnant women were monitored and received IPTp according to the national protocol of the Malagasy Ministry of Health.

### 2.7. Health System and Sociocultural Context

Access to healthcare in the Ambatoboeny District is significantly hindered by economic and sociocultural factors. Many people in the region live below the poverty line, and a substantial portion of the population struggles with the inability to afford healthcare services. As a result, individuals often delay seeking medical help until their health problems become serious. Traditional methods, such as beliefs or folk remedies, are used as long as they seem to provide relief, with people turning to hospital care only when these methods fail. This reluctance to seek medical care is often due to the high costs associated with hospital visits and specialist treatments [11]. In addition, traditional beliefs about the causes of illness play a significant role in healthcare-seeking behaviour. Many people in the region attribute illnesses to spiritual or ancestral causes, leading them to consult traditional healers instead of healthcare professionals. Such practices can delay access to effective biomedical treatments and worsen health conditions before hospital care is sought. Overcoming these barriers, both financial and cultural, is essential for improving healthcare accessibility and quality in the region [12].

## 3. Results

### 3.1. Study Population Characteristics

A total of 3678 patient admissions were recorded at Médicale Clinique BEYZYM in Manerinerina, Madagascar. The median age of patients was 19.5 years (IQR: 7–42), with the majority belonging to the 25–59-year age group (26.48%). Females constituted 54% of the study population. The vast majority of patients (87.19%) resided in the Ambatoboeny district. Detailed sociodemographic characteristics are presented in Table 2.

### 3.2. Patterns of Morbidity

(a) Overall Morbidity and Disease Distribution

**Figure 3:**
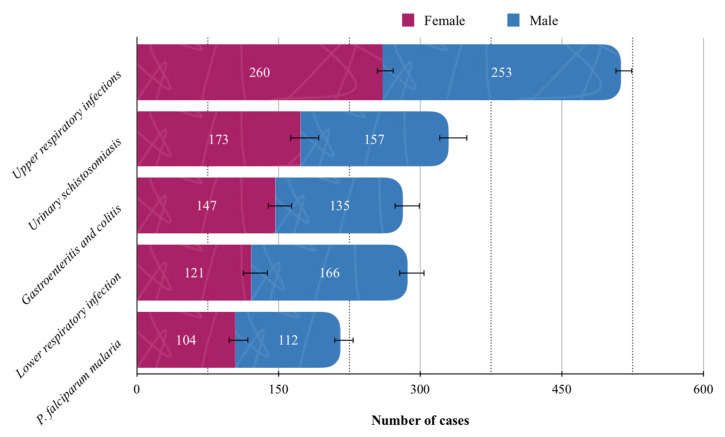
Top five most common individual conditions in the study population. Solid lines standard errors, and dashed vertical lines are auxiliary lines to help visualize the number of cases.

The most common reasons for admission were infectious and parasitic diseases (35.75%, 95%CI: 34.20–37.30), respiratory diseases (22.73%, 95% CI: 21.38–24.08), and diseases of the genitourinary system (13.95%, 95%CI: 12.83–15.07), collectively accounting for 72.43% of all recorded cases (Table 3). The five most prevalent disease categories in the overall population are illustrated in Figure 3, with upper respiratory infections being the most frequent (n = 513, 13.95%; 95% CI: 12.83–15.07). Causes of admission presented in Table 3 were grouped according to the main ICD-11 chapters, while Figure 3 illustrates the most frequent individual conditions within these categories.

(b) Sex-Specific Morbidity

**Figure 4:**
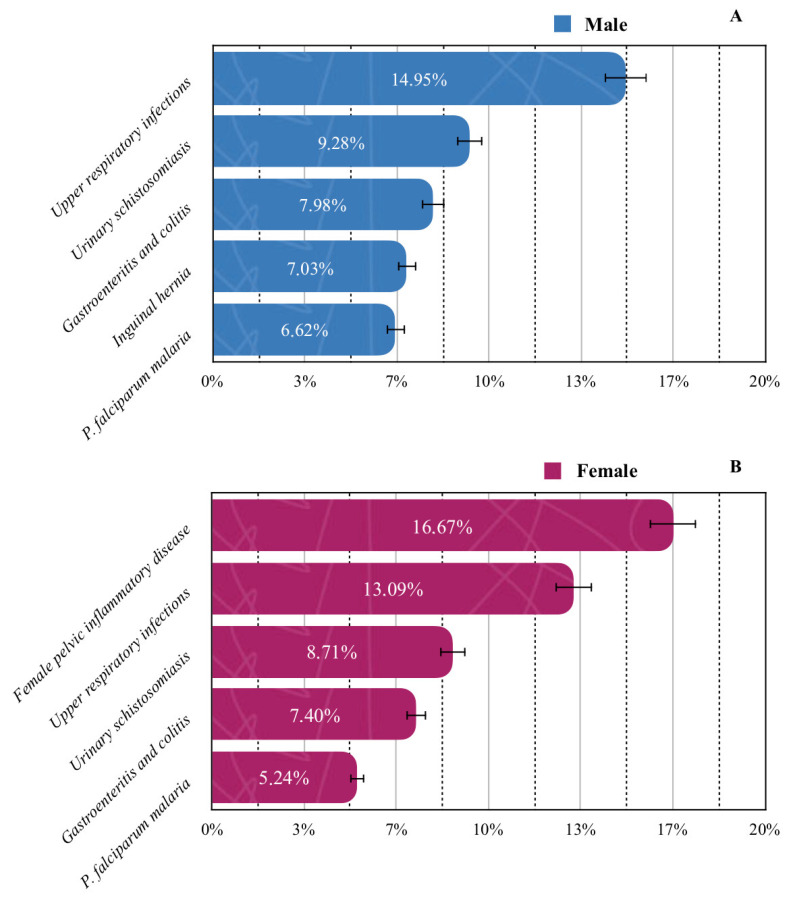
Prevalence of the five most common diseases by gender: (**A**) male; (**B**) female. Solid lines standard errors, and dashed vertical lines are auxiliary lines to help visualize the number of cases.

When stratified by sex, upper respiratory infections were most prevalent among males (14.95%), whereas female pelvic inflammatory disease predominated in females (16.67%) (Figure 4). Pregnancy-related conditions were exclusively observed in the female population and were therefore excluded from overall prevalence analyses.

(c) Age-Specific Morbidity

**Figure 5:**
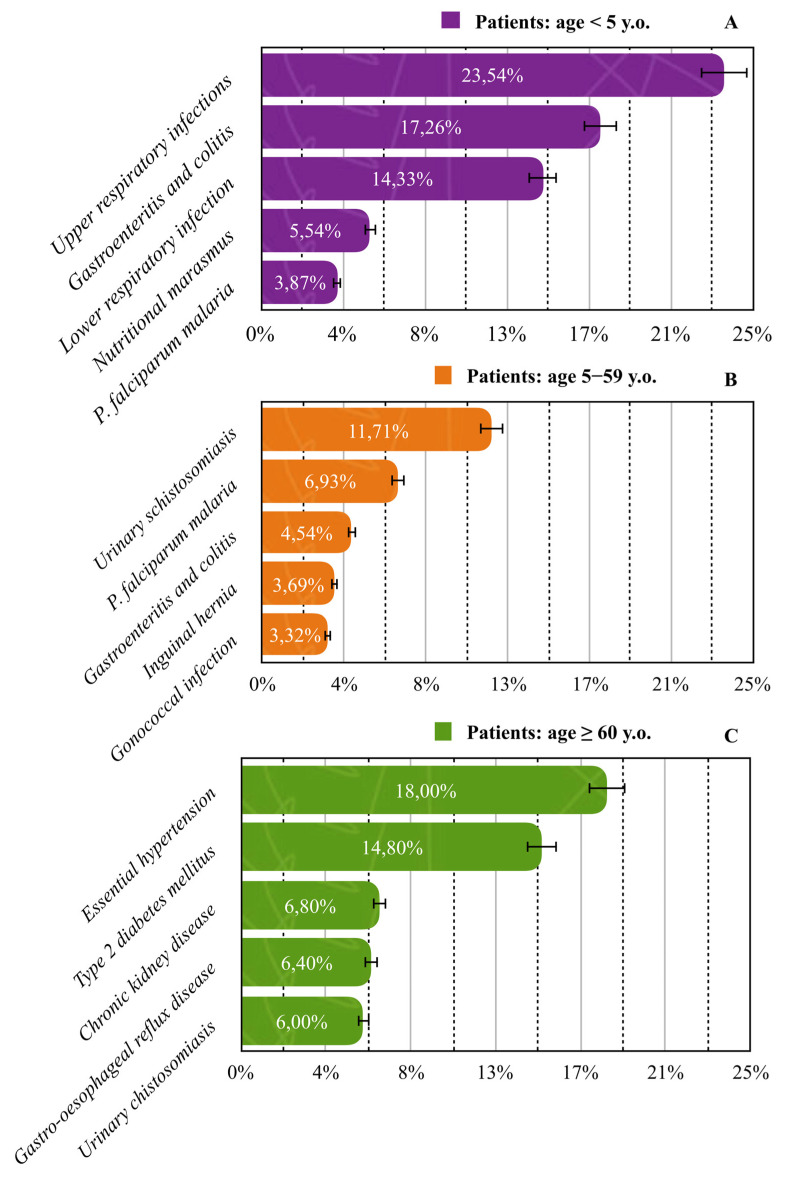
Prevalence of the five most common diseases across age groups: (**A**) <5 years; (**B**) 5–59 years; (**C**) >60 years. Solid lines standard errors, and dashed vertical lines are auxiliary lines to help visualize the number of cases.

In children under five years of age, upper respiratory infections were the leading cause of admission (23.54%). Among patients aged 5–59 years, schistosomiasis caused by *S. haematobium* was most common (11.71%), while in the ≥60-year group, essential (primary) hypertension accounted for the highest proportion of cases (18.0%). All most common diseases across age groups are presented in Figure 5.

Specific Disease Patterns

**Figure 6:**
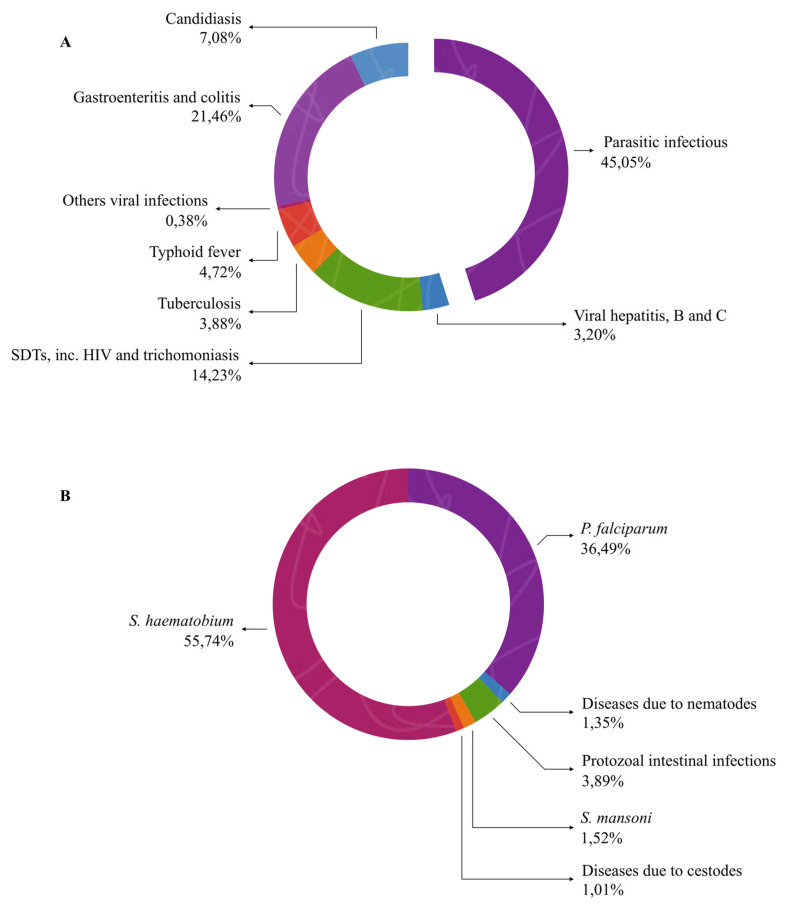
Proportional distribution of infectious and parasitic diseases (**A**) and detailed classification of parasitic diseases (**B**).

The distribution of diseases among patients admitted to Médicale Clinique BEYZYM reveals a significant predominance of infectious and parasitic diseases. As shown in Figure 6A, infectious and parasitic diseases accounted for a large proportion of the admissions, with parasitic diseases alone representing 45.05% of all cases. Among parasitic diseases, the most prevalent were those caused by *S. haematobium* (55.74%) and *P. falciparum* (46.79%), as illustrated in Figure 6B, which presents the detailed classification of parasitic diseases. The remaining cases were attributed to various intestinal parasites. This pattern highlights the substantial burden of parasitic diseases in the region, with a predominance of schistosomiasis and malaria.

### 3.3. Statistical Test of the Relationship Between Demographic Variables and Morbidity

In order to investigate the influence of demographic variables—sex and age—on morbidity among patients reporting to the healthcare facility in the Ambatoboeny district (Boeny region, Madagascar), an analysis was conducted based on admission data categorised by age groups (0–14, 15–24, 25–59, 60+) and sex (male and female). The data were grouped into 14 major disease categories according to the ICD-11 classification. The category “Pregnancy, childbirth and the puerperium” was excluded from the analysis, as it exclusively applies to females and could bias the overall assessment of sex-related differences in morbidity. To assess the association between sex and disease categories, a chi-squared test of independence was performed with a 95% confidence level (α = 0.05). The analysis revealed a statistically significant difference in the distribution of morbidity between males and females (χ^2^ = 364.65; df = 13; *p* < 0.001). Consequently, the null hypothesis of no association was rejected, indicating that sex has an effect on the pattern of morbidity in the studied population. To estimate the strength of this association, Cramér’s V was calculated, yielding a value of 0.315, which indicates a moderate relationship between sex and the distribution of disease diagnoses. Although confidence intervals are not routinely reported for Cramér’s V, the effect size was interpreted together with the significant chi-squared test result, indicating a moderate relationship between sex and the distribution of disease diagnoses.

Furthermore, to evaluate whether age influences the overall level of morbidity, a Spearman’s rank correlation test was conducted, treating age as an ordinal variable based on the four predefined age intervals. The analysis was carried out with a 95% confidence level (α = 0.05). The Spearman correlation coefficient was ρ = −0.80, but with a *p*-value of 0.20, this result was not statistically significant. Thus, no significant correlation was found between age and the number of disease cases in the study sample (Table 4).

## 4. Discussion

This study demonstrates that infectious and parasitic diseases are the primary causes of hospital admissions in the Ambatoboeny district, accounting for 35.75% of all cases (95% CI: 34.20–37.30), followed by respiratory system diseases (22.73%; 95% CI: 21.38–24.08) and genitourinary system diseases (13.95%; 95% CI: 12.83–15.07). Together, these three categories represent over 72% of the recorded cases. The five most common specific diagnoses were upper respiratory tract infections, urinary schistosomiasis, gastroenteritis and colitis, lower respiratory tract infections, and *P. falciparum* malaria. These findings are consistent with national statistics from WHO, UNICEF, and the Ministry of Public Health in Madagascar, which identify infectious diseases—including malaria, respiratory infections, and waterborne illnesses—as the leading causes of morbidity and mortality in the country [2,13,14]. A broader sub-Saharan perspective confirms this trend. For instance, a meta-analysis by Etyang et al. (2013) found that among 86,307 hospital admissions in sub-Saharan Africa, the leading causes were infectious and parasitic diseases (19.8%), respiratory diseases (16.2%), and circulatory diseases (11.3%) [15]. Similar patterns have been reported in rural Mozambique [16], Tanzania [17,18,19], and Uganda [20], where communicable diseases dominate, particularly in low-resource settings with limited access to healthcare and sanitation. Data from other African studies reinforce the dominance of infectious pathologies. In Côte d’Ivoire, a retrospective analysis of 300 pediatric cases revealed that infectious and parasitic diseases accounted for 43.7% of hospital stays, with *P. falciparum* malaria being the most frequent diagnosis (24.3%) [21]. Similarly, Kannan (2016) highlighted trauma, gastrointestinal, and infectious diseases as the most frequent emergency presentations in Madagascar [22], while Digbeu (2004) and Dodo (2024) emphasised both a high prevalence and clinical severity of urinary and parasitic infections, particularly among patients with co-morbidities such as chronic kidney disease [21,23]. Focusing specifically on the Ambatoboeny district, parasitic infections accounted for 45.05% of all infectious disease admissions. Within this group, schistosomiasis caused by *S. haematobium* and malaria due to *P. falciparum* were the most prevalent. Schistosomiasis was the leading diagnosis among patients aged 5–59 years (11.71%), followed by malaria (6.93%). *S. haematobium* consistently ranked among the top five diagnoses across most age and sex groups, excluding children under five. While partial, age-related immunity against malaria may develop after repeated exposures, long-term protective immunity does not occur in schistosomiasis, where infections frequently follow the cycle of infected–treated–(re)infected. This lack of sustained immunity explains the wide age distribution of schistosomiasis observed in our study and reflects global findings highlighting its role as a persistent neglected tropical disease [24,25]. The predominance of these two parasitic diseases is also shaped by local environmental conditions. Warm temperatures and seasonal rainfall in the Boeny region create favorable conditions for both *Anopheles* mosquitoes, the primary vectors of *P. falciparum*, and freshwater snails of the genus *Bulinus*, which serve as the intermediate hosts of *S. haematobium*. The widespread presence of rice fields, ponds, and irrigation canals provides ideal breeding sites for these vectors and intermediate hosts, thereby facilitating ongoing transmission throughout the community [26,27].

This localized morbidity pattern is well supported by the literature. Studies conducted in the neighbouring Ankazoborina district identified *S. haematobium* as the dominant parasitic infection among adults [28]. Rasoamanamihaja et al. further highlighted the urgency of implementing targeted public health measures aligned with WHO’s 2021–2030 NTD roadmap [29]. Wilczyńska et al. reported alarmingly high *S. haematobium* prevalence in Ambatoboeny, with infection rates reaching 67.6% among 170 children [30]. Greigert et al. also confirmed widespread intestinal protozoal infections in both rural and urban Madagascar, identifying poor sanitation as a key factor responsible for the disease transmission [31]. Similarly, Richert W. et al. found that in a sample of 208 individuals, potentially pathogenic stramenopiles *Blastocystis* spp. (32.0%) and the protozoan *Giardia intestinalis* (20.5%) were responsible for the majority of infections [32]. In addition to schistosomiasis, the continued transmission of *P. falciparum* malaria remains a critical concern. According to WorldData (2024), malaria is among the leading causes of death in Madagascar [1]. Mapping by Nguyen et al. confirms that Ambatoboeny is classified as a high-risk area for seasonal malaria transmission [33].

The age-specific distribution of diseases offers further insight. Among children under five, upper respiratory infections were the leading cause of admissions (23.54%), followed by gastroenteritis (17.26%), lower respiratory tract infections (14.33%), and malaria (3.87%). Notably, nutritional marasmus accounted for 5.54% of hospitalisations in this group, highlighting the persistent problem of early childhood malnutrition. This finding aligns with national estimates indicating that 38.6% of Malagasy children under five are stunted and 7.2% are wasted [34]. Malnutrition in this age group is closely linked with poverty, recurrent infections, and parasitic diseases. A study by Alfano found that 100% of school-aged children enrolled in a nutritional recovery programme in Madagascar were undernourished, with 32.6% experiencing stunting. Additionally, nearly all participants were infected with *G. intestinalis* (93.9%) and *Trichuris trichiura* (28.6%), directly linking undernutrition to parasitic burden [26]. This interplay between infectious diseases and poor nutritional status exacerbates morbidity and impairs child development. International data support these findings: UNICEF, WHO, and the World Bank report that nearly half of all children affected by wasting or stunting live in Africa, and these conditions contribute to long-term cognitive impairment, reduced educational attainment, and lower economic productivity in adulthood [35]. Among patients aged ≥60 years, NCDs constituted the predominant morbidity pattern, with essential hypertension (18%) and type 2 diabetes mellitus (14.8%) being the most frequent. Chronic kidney disease (6.8%) and gastroesophageal reflux disease (6.4%) were also notable, while urinary schistosomiasis (6%) —though parasitic—remains a persistent burden in older populations. This age-stratified burden reflects ongoing exposure to infectious agents alongside an emerging pattern of chronic diseases, a dual challenge commonly seen in sub-Saharan Africa [36]. These findings are in line with recent studies from Madagascar and the region, which document a growing epidemiological transition toward NCDs in aging populations. Dodo et al. reported that urinary tract infections were more frequent and severe in patients with chronic kidney disease, with older age and male sex identified as major predictors of complications and antibiotic resistance [23]. Similarly, Randrianarisoa reported a high prevalence of dyslipidemia (68.2%), reaching 70.6% among individuals over 65 years, with the most common lipid abnormalities being hypercholesterolemia and hypo-HDL cholesterolemia. These were significantly associated with women, diabetes, and morbid obesity, underscoring the need for integrated metabolic risk management in elderly cohorts [37]. Furthermore, Raharinavalona found that hypovitaminosis D was highly prevalent among patients with type 2 diabetes (66%), particularly those aged ≥70 years, and was strongly associated with multiple cardiovascular risk factors, including hypertension (OR = 8.77), dyslipidemia (OR = 8.05), carotid atherosclerosis (OR = 2.96), and microalbuminuria (OR = 2.95) [38]. These associations underscore the complex interplay between aging, metabolic dysregulation, and micronutrient deficiencies. Additionally, stroke remains a major public health concern in sub-Saharan Africa and has reached an alarmingly high prevalence in Madagascar (48.17 per 1000), the highest in the region according to Abissegue. Hypertension, diabetes, dyslipidemia, and cardiovascular comorbidities were identified as the main risk factors [39]. This convergence of chronic metabolic and vascular conditions in older populations reflects the growing double burden of disease—infectious and non-communicable—facing low- and middle-income countries. The data from Ambatoboeny thus present a microcosm of this broader trend, underscoring the urgency of integrated, age-sensitive public health strategies.

## 5. Limitations

This study presents several important limitations which must be considered when interpreting the findings. First, the retrospective design relied on existing medical records, which were sometimes incomplete or inconsistent. This may have introduced information bias, particularly in the documentation of symptoms and diagnostic precision. Although incomplete records were excluded from the analysis, many cases were managed symptomatically without a confirmed diagnosis, resulting in 212 patients being classified under ICD-11 code R (Symptoms, signs and abnormal clinical and laboratory findings, not elsewhere classified).

Second, the diagnostic capacity of the facility was limited by the availability of laboratory and imaging equipment. Intestinal parasitic infections may have been under diagnosed due to reliance on basic stool examination techniques, such as direct smear and iodine staining, without more sensitive methods like concentration or flotation techniques. In particular, while helminths could usually be detected with reasonable sensitivity, the accurate diagnosis of protozoal infections (e.g., *Giardia intestinalis*, *Entamoeba histolytica*) was far more problematic. Laboratory reports often referred only to “protozoa” or “*Entamoeba* sp.” without species-level confirmation. For consistency, such cases were classified under gastroenteritis of infectious origin (ICD-11: 1A00–1A0Z) based on clinical presentation and supporting smear results. This classification approach may account for some apparent inconsistencies in the distribution of gastrointestinal infections in the dataset.

Additionally, the majority of physicians in Madagascar do not hold clinical specializations, and their medical training is predominantly undergraduate. As a result, diagnoses may have been overly generalized or simplified, particularly for conditions like gastroenteritis and colitis, which were frequently treated empirically without further diagnostic differentiation. This could have led to underrepresentation of specific infectious or parasitic diseases, including those caused by *Entamoeba* spp. and other protozoa.

Furthermore, since the study was conducted in a single secondary-level referral facility, the results may not be generalizable to the entire Ambatoboeny District population, particularly those living in remote rural areas with limited access to hospital-based healthcare. Health-seeking behavior, shaped by sociocultural beliefs and financial barriers, likely influenced the patient profile, favoring more severe or treatment-resistant cases.

Although ICD-11 was used as the diagnostic framework, its application in a field setting posed challenges. Newly introduced categories, such as “Conditions related to sexual health,” had not yet been adopted in local reporting systems. Consequently, sexually transmitted infections were pragmatically classified under “Certain infectious or parasitic diseases” (code A), in accordance with existing national practice.

Hospital records did not include data on patient mortality or length of hospital stay. Fatal outcomes were often omitted from documentation. Lastly, demographic data were limited to age, sex, and place of residence. Socioeconomic indicators such as income level, educational background, and occupation were not recorded, constraining the depth of the sociodemographic analysis.

## 6. Conclusions

This study addresses a critical gap in demographic and clinical data by presenting a detailed profile of disease burden in the Ambatoboeny district—an underrepresented region in public health research. The findings underscore that infectious and parasitic diseases remain the leading causes of hospital admissions, particularly among children and young adults. However, the data also reveal a significant and rising burden of NCDs among older adults, including hypertension, diabetes, and chronic kidney disease. This dual burden of disease poses a major challenge for frontline healthcare providers in Ambatoboeny and similar low-resource settings. Medical personnel must navigate both communicable and non-communicable conditions, often without access to comprehensive diagnostic tools. This requires not only broad clinical knowledge and flexibility, but also a high degree of diagnostic intuition and adaptability in environments with limited laboratory capacity. The implications of this study extend beyond academia. Policymakers, public health authorities, and humanitarian organizations should consider these findings when allocating resources and designing interventions in Boeny and comparable regions.

Attention should also be given to sex-specific disparities, as women often face disproportionate barriers to healthcare access, including limited availability of gynecological services and higher exposure to parasitic infections through daily household and agricultural activities. In addition, effective public health strategies must also account for environmental and sanitation management, given their close link with infectious disease transmission. Special emphasis should be placed on training and deploying health professionals with a background in both tropical medicine and chronic disease management. Such preparation would better equip medical teams for the complexity of real-world practice in Madagascar’s peripheral districts.

Ultimately, the data presented here offer a foundation for evidence-based planning, highlighting the urgent need for integrated, age- and context-sensitive strategies to reduce disease burden and strengthen the resilience of local health systems.

## Figures and Tables

**Table 1:** ICD-11 Diagnostic Categories Excluded from the Analysis and Reasons for Omission.

ICD-11 Category	Reason for Exclusion *
Mental, behavioural or neurodevelopmental disorders	Facility lacks psychiatric services
Congenital malformations, deformations and chromosomal abnormalities	Cases referred to tertiary-level hospitals
Diseases of the musculoskeletal system and connective tissue	Rarely recorded; limited diagnostic tools
Diseases of the nervous system	Poor documentation or underreporting
Diseases of the oral cavity and salivary glands	Not managed by the facility
Conditions related to sexual health	Not systematically recorded **
Developmental anomalies and social problems	Not within the scope of hospital data collection

* These exclusions reflect both the clinical profile of the facility and the availability of data in the hospital records during the study period. ** In this analysis, sexually transmitted diseases (STDs) were classified under the category of “Infectious and parasitic diseases” (Chapter 1, codes A) according to the ICD-11 classification system. Although ICD-11 introduces a broader framework under “Conditions related to sexual health” (code QA), which encompasses both biomedical and psychosocial dimensions of sexual health, this category was not applied in the present study. The decision was based on the practical consideration that the QA category remains relatively new and is not yet widely adopted in public health reporting systems in Madagascar. Consequently, due to limited availability of disaggregated data and prevailing national classification practices, all STD-related diagnoses were consistently categorised within the infectious disease framework.

**Table 2:** Sociodemographic characteristics of patients (*n* = 3678).

Variables	*n* (%)
**Gender**	
Male	1692 (46.00)
Female	1986 (54.00)
**Age**	
0–4	959 (26.07)
5–14	733 (19.93)
15–24	762 (20.72)
25–60	974 (26.48)
60+	250 (6.80)
**Residence**	
District Ambatoboeny	3207 (87.19)
District Mampikony	352 (9.57)
Other	119 (3.24)

**Table 3:** Reasons for admission by ICD-11 * (*n* = 3678).

ICD Code	Reason for Admission	*n* (%) [95% CI]		
		Total	Male	Female
A or B	Certain infectious and parasitic diseases	1315 (35.75) [34.20; 37.30]	627 (47.68) [2.70; 44.98]	688 (52.32) [49.62; 55.02]
C or D	Neoplasms	29 (0.79) [0.50; 1.07]	1 (3.45) [0.00; 10.09]	28 (96.55) [89.91; 100.00]
D	Diseases of the blood	15 (0.41) [0.20; 0.61]	10 (66.67) [42.81; 90.52]	5 (33.33) [9.48; 57.19]
E	Endocrine, nutritional and metabolic diseases	116 (3.15) [2.59; 3.72]	55 (47.41) [38.33; 56.50]	61 (52.59) [43.50; 61,67]
H	Diseases of the eye and adnexa	34 (0.92) [0.62; 1.23]	18 (52.94) [36.16; 69.72]	16 (47.06) [30.28; 63.84]
H	Diseases of the ear	49 (1.33) [0.96; 1.70]	26 (53.06) [39.09; 67.03]	23 (46.94) [32.97; 60.91]
I	Diseases of the circulatory system	103 (2.80) [2.27; 3.33]	74 (71.84) [63.16; 80.53]	29 (28.16) [19.47; 36.84]
J	Diseases of the respiratory system	836 (22.73) [21.38; 24.08]	412 (49.28) [45.89; 52.67]	424 (50.72) [47.33; 54.11]
K	Diseases of the digestive system	214 (5.82) [5.06; 6.57]	177 (82.71) [77.64; 87.78]	37 (17.29) [12.22; 22,36]
L	Diseases of the skin	65 (1.77) [1.34; 2.19]	29 (44.62) [32.53; 56.70]	36 (55.38) [43.30; 67,47]
N	Diseases of the genitourinary system	513 (13.95) [12.83; 15.07]	100 (19.49) [16.07; 22.92]	413 (80.51) [77.08; 83.93]
O	Pregnancy, childbirth and the puerperium	79 (2.15) [1.68; 2.62]	0 (0.00) [0.00; 0.00]	79 (100.00) [100.00; 100.00]
P	Certain conditions originating in the perinatal period	33 (0.90) [0,59; 1.20]	19 (57.58) [40.71; 74.44]	14 (42.42) [25.56; 59.29]
S or T	Injury and poisoning	65 (1.77) [1.34; 2.19]	40 (61.54) [49.71; 73.37]	25 (38.46) [26.63; 50.29]
R	Symptoms, signs and abnormal clinical and laboratory findings, not elsewhere classified	212 (5.76) [5.01; 6.52]	104 (49.06) [42.33; 55.79]	108 (50.94) [44.21; 57.67]

* ICD—International Classification of Disease; categorized using Version 11.0 of the World Health Organization’s ICD coding system: https://icd.who.int/en/ (accessed on 25 April 2025).

**Table 4:** Statistical analysis of sex and age in relation to morbidity.

Statistical Test	Test Statistic Value	*p*-Value	Conclusion
χ^2^ (sex vs. disease categories, excl. pregnancy)	364.65 (df = 13)	*p* < 0.001	Statistically significant association
Spearman’s correlation (age vs. morbidity)	−0.80	0.20	No statistically significant correlation

## Data Availability

The data presented in this study are available on request from the corresponding author.
